# Adolescents’ experiences of psychological treatment for gaming disorder: a qualitative study

**DOI:** 10.3389/fpsyt.2025.1601851

**Published:** 2025-06-03

**Authors:** Per Bore, Jenny Leo, Lars Garpenhag, Emma Claesdotter-Knutsson

**Affiliations:** ^1^ Department of Clinical Sciences Lund, Faculty of Medicine, Lund University, Lund, ;Sweden; ^2^ Malmö Addiction Center, Malmö, ;Sweden; ^3^ Child and Adolescent Psychiatry, Lund, ;Sweden

**Keywords:** gaming disorder, internet gaming disorder, adolescents, youth, treatment seeking, qualitative analysis, clinical sample

## Abstract

**Background:**

Gaming disorder is a recently recognized psychiatric condition and a growing public health concern, particularly among adolescents. Despite this, there is limited research on what kind of treatment they need. Existing studies are mostly quantitative and offer limited insight into adolescents’ lived experiences.

**Aim:**

The aim of this qualitative study is to explore how adolescent patients perceive their gaming as a problem and their experiences of psychological treatment for gaming disorder.

**Method:**

We used a qualitative descriptive approach and conducted semi-structed interviews with eight male patients (aged 13–19) about their experiences of psychological treatment for gaming disorder at a specialized clinic. The treatment is a combination of cognitive-behavioral therapy (CBT) and family therapy. The interviews were analyzed using thematic analysis.

**Results:**

Participants generally reported positive treatment experiences, especially the value of combining family therapy with individual CBT. They appreciated the broad focus of the treatment, which addressed not only gaming but also problems in other life areas such as school, sleep, and family relationships. Notably, most did not describe gaming as their main problem, but they connected their gaming to difficulties in other areas of life.

**Conclusion:**

These findings suggest that effective treatment for gaming disorder should address the broader psychosocial context in which gaming occurs. Patients do not always view gaming as their primary problem, and clinicians should be cautious about framing it as such. Instead, gaming should be explored in connection with other life difficulties. It is helpful when clinicians demonstrate knowledge about gaming and avoid coming across as critical of the gaming. Integrating family therapy into CBT-based interventions appears clinically valuable and warrants further exploration.

## 1 Introduction

Gaming disorder is defined as gaming behavior characterized by diminished control over gaming, prioritizing gaming over other activities, continuation of gaming despite negative consequences, and gaming causing significant impairments in important areas of functioning (1). Officially recognized as a psychiatric disorder in 2018, it was included in the 11th revision of the International Classification of Diseases (ICD-11). There is a discussion in the research community regarding gaming disorder as a formal psychiatric diagnosis. Some critics argue that the establishment of the diagnosis went too fast, that it is not scientific underbuilt, driven by moral panic, and risks pathologizing normal youth behaviors (2–4). Others argue that many studies do exist and establishing gaming disorder as a diagnosis encourages further research that is needed in this field (5, 6).

Gaming is a popular leisure activity among adolescents in Sweden (7). Healthcare, social service, and school professionals in Sweden are increasingly concerned about problematic gaming and point to the need for more knowledge on the issue (8). Despite the formal recognition as a diagnosis, there is a lack of knowledge about gaming disorder, who seeks treatment for it, and what care they need. Globally, the prevalence of gaming disorder has been estimated at around 2% (9). Younger individuals and males are more likely to engage in gaming, and boys are overrepresented among those diagnosed with gaming disorder (9). Gaming disorder is associated with other psychiatric conditions and is linked to sleep problems, academic problems and loneliness (10–12). Research also shows that individuals with gaming disorder is more prone to experience family-related challenges, such as more conflict, poorer family climates, and less family support (13–15).

Currently, there is no widely accepted treatment for gaming disorder. Most existing studies have focused on cognitive-behavioral therapy (CBT), which has shown promising results (16–18). CBT is based on the idea that problematic behaviors are maintained by maladaptive thoughts, difficulties in emotional regulation, and avoidance patterns. In the context of gaming disorder, CBT typically focus on identifying and challenging maladaptive thoughts, developing alternative coping strategies, improving emotion regulation, training social skills, managing gaming-related triggers, and establishing new behavioral routines (19, 20). A randomized controlled trial (RCT) from our research group looked at Relapse prevention as a possible treatment for gaming disorder (21). Relapse prevention reduced symptoms of gaming disorder, but the family component was missing and might have improved the effectiveness of the treatment (22, 23). A review by Kim, Lee (18) suggests that combining CBT with family therapy may further improve treatment outcomes. Bonnaire, Liddle (14) argue that younger individuals with gaming disorder often rely on family members for support and structure, which highlights the potential benefits of family-based interventions. For example, CBT combined with parent psychoeducation was evaluated in a RCT with 31 adolescents and showed positive outcomes in reducing gaming disorder symptoms, improved comorbid symptoms and family relationships (24). Another RCT compared Multidimensional Family Therapy with standard family therapy in a sample of 42 adolescents, while no significant differences were found between the groups, both groups showed symptom reduction (25). A third RCT tested a group-based training parent training with 76 parents of adolescents with internet-related disorders. Parent-reported gaming disorder symptoms improved for at-risk users, though not for those with more severe, pathological use (26). Finally, an online parent intervention study for parents of school-aged children with Internet gaming disorder and/or smartphone addiction showed promising results in a sample of 153 parent who completed. Among children with high levels of Internet Gaming Disorder, 60% showed symptom reduction following the intervention (27). Together, these findings suggest that both CBT and family-based interventions hold promise for treating gaming disorder, and that combining these approaches may be particularly effective.

More research is needed on psychological treatments for gaming disorder, particularly family-based interventions. Since most existing studies are quantitative, there is also a need for studies exploring patients’ own perspectives on treatment, which are important for directing the continued development of effective treatments (2). Gaining deeper insight into the experiences of patients, especially adolescents, may help identify mechanisms of change and support the creation of more effective treatments. The current study aims to address some of the gaps in previous research by exploring how adolescent patients perceive their gaming behavior and their experiences of psychological treatment for gaming disorder. Using a qualitative approach, the study focuses on the following research questions: (1) Do adolescent patients seeking treatment for gaming disorder perceive their gaming as a problem, and if so, how? (2) How do they experience undergoing treatment at a specialized clinic for gaming disorder? (3) How do they experience individual therapy sessions compared to family sessions within treatment for gaming disorder?

## 2 Material and methods

### 2.1 Study design

This study used a qualitative design to explore patients’ experiences of undergoing psychological treatment for gaming disorder. Data were collected through semi-structured interviews, and analysis followed a qualitative descriptive approach using thematic analysis, focusing on summarizing and categorizing participant responses. The qualitative descriptive approach was chosen to provide a comprehensive summary of patient experiences while remaining close to their actual descriptions (28, 29).

### 2.2 Study participants and recruitment

Participants were recruited through purposive sampling from a specialized clinic offering psychological treatment for gaming disorder. Eligible participants were aged 13–19 years and had either completed or were in the final phase of the treatment. A list of all possible interview candidates was created (N=25). The aim was to include both male and female participants, however, as there was only one female on the list of potential candidates, we chose to exclude her to reduce the heterogeneity of the sample. Two participants were excluded because only their parents had participated in the treatment, and the adolescents themselves had no direct contact with the therapist. An additional three candidates were excluded, due to a combination of their young age (13–14 years) and other factors, indicating they were too vulnerable to participate in an interview with an unfamiliar interviewer. This left 19 eligible participants, who were contacted to ask if they were interested in participating in the study. Of those three did not respond when called by telephone or contacted with text messages. Seven eligible participants declined to participate when asked. Nine patients initially agreed to participate, but one did not attend the scheduled interview. The final sample thus consisted of eight male participants who completed the interview process. In the end, we interviewed all eligible patients who agreed to participate at the start of the study. For information about the participans, see **Table 1**.

**Table 1:** Description of participants.

Participant Nr	Age, at interview	Comorbid psychiatric diagnosis	Weekly game time, before treatment	Individual sessions	Family sessions	Games
P1	18	–	50h	X	X	Counter-Strike
P2	19	–	60h	X	X	FIFA and Counter-Strike
P3	18	Autism (F840), ADD (F900C)	42h	X		League of Legends
P4	17	Feeding disorder (F982), Autism (F840), ADD (F900C)	64h	X	X	Entropy: Zero 2 and Man of War
P5	13		10h	X	X	Fortnite
P6	18	Mixed anxiety and depressive disorder (F412)	70h+	X	X	Counter-Strike
P7	17	–	42h	X	X	Fortnite
P8	15	ADHD (F900B)	56h	X	X	Rocket League

### 2.3 Treatment description

The psychological treatment provided at the specialized clinic was developed specifically for gaming disorder and is described in more detail, including its development process, in a previously published protocol (47). The treatment is module-based and the modules consists of interventions from CBT and family therapy. It is tailored to each participant, with therapists selecting relevant modules based on clinical assessment of the adolescent’s age, family situation, level of functioning, and treatment goals. Treatment generally aims at 15 treatment sessions covering one to six modules, although this may be adjusted based on individual needs.

The treatment includes ten CBT-based individual modules and seven FT modules. The individual sessions aim to help participants reduce problematic gaming, engage in alternative activities, and develop new coping strategies. Modules cover areas such as behavioral activation, daily structure, psychoeducation on sleep, nutrition and exercise, impulse control, emotion regulation, working with thoughts, social anxiety, procrastination, conflict management and problem-solving. The family therapy modules include sessions with the entire family, the parents alone, or the adolescent alone, in most treatment it is a combination of these. The family therapy modules can be delivered in sessions with the entire family, the parents alone, or the adolescent alone, and in most cases, a combination of these formats is used. These modules aim to reduce conflict, increase engagement in other activities, and increase parental support. Topics include psychoeducation about gaming, alternative activities to gaming, increasing positive interactions, balancing demands and support, making agreements, setting boundaries with emotional validation, and conflict management.

While some sessions are clearly structured as either individual or family-based, the treatment encourages integration, allowing therapists to combine CBT and family therapy strategies. For example, emotion regulation skills from CBT may be reinforced through family sessions that focus on validating the adolescent’s emotional experiences and reducing conflict at home. Overall, the treatment emphasizes understanding the function of gaming in each participant’s life, identifying why they struggle to balance gaming with other responsibilities, and supporting them in re-engaging with school, work, and daily activities.

On average, participants received a total of 18.75 sessions (range: 7-28). For most, the first three sessions were dedicated to assessment and treatment planning, with the remaining were treatment sessions. All but one participant received both CBT and family therapy sessions; one participant received only individual CBT sessions. The ratio between CBT and family sessions varied across the participants, with an average of 12.5 family sessions (range: 0–26) and 6.25 individual CBT sessions (range: 2–23). Missed sessions occurred in all treatments, with an average of 4 missed sessions per participant (range: 1–6). Of the eight treatments, two ended earlier than initially planned, after 7 and 8 sessions, respectively.

### 2.4 Data collection

Interviews were conducted after treatment completion or in the final phase of treatment. All interviews were conducted by the second author, who was not affiliated with the clinic and had no prior contact with the participants, The interviews were conducted face-to-face or via videoconferencing, depending on participant preference. The interviews followed an interview-guide (see supplement) with open-ended questions and follow-up questions. The average interview length was 34 minutes. All interviews were audio-recorded and transcribed verbatim. No notable differences in length or content were observed between in-person and videoconferencing interviews.

### 2.5 Data analysis

The study followed a qualitative descriptive research design, as described by Sandelowski (28) and Villamin, Lopez (30). The data were analyzed using thematic analysis (31). The aim was to remain close to the data, providing straightforward descriptions, and summarizing participant experiences. To analyze the data, the first and second authors independently read all transcripts to familiarize themselves with the content. They then coded the first four interviews separately, identifying key descriptive elements. Afterward, the first, second, and third authors met to discuss and refine the coding structure. Using this refined code structure, the first and second authors analyzed the remaining four interviews separately (they were also allowed to add new codes). A meeting with the third author was held to confirm the coding structure. To ensure consistency and accuracy, the first and second authors re-analyzed all interviews separately using the final coding framework. Lastly, the first, second, and third authors met again to finalize the descriptive categories and summarize the findings.

All participants who were available at the starting point of the study were included in the sample. During the process of analyzing the data, saturation was continuously assessed, and as the existing data provided was determined sufficient to address the research questions, no further participants were invited to the study even as new patients entered treatment.

### 2.6 Ethical considerations

The study was approved by the Swedish Ethical Review Authority (DNr: 2021-05923–01 and 2023-06393-02). Written informed consent was obtained from participants aged 15 years and older. For those aged 13–14 years, consent was obtained from their legal guardians. Data were pseudonymized to protect participant identities, with participants labeled as Participant 1 to 8 (P1-P8) and all transcripts were stored securely. Participants were informed that participation was voluntary and that they could withdraw at any time. Given the nature of the study, participants were provided with information on support services in case discussing their experiences caused any distress. The study followed to the ethical principles outlined in the Declaration of Helsinki.

## 3 Results

The analysis of the interviews identified six main categories that reflect male adolescents’ perceptions of gaming as a problem and their experiences of psychological treatment for gaming disorder. The first category, *Perception of gaming as a problem*, explores how participants viewed their gaming behavior and the factors that contributed to it becoming problematic. *Help-seeking and motivation for treatment* focuses on what motivated participants or their families to seek help and how motivation changed over time. In *Experiences and reflections on treatment*, participants shared their overall satisfaction, suggestions for improvement, and reflections on the treatment’s focus. *Changes following treatment* describes perceived improvements in gaming behavior, school functioning, daily routines, and family relationships. The category *The therapeutic relationship and the therapist’s knowledge of gaming* highlights how the therapeutic relationship and the therapist’s familiarity with gaming effects participants’ experiences. Finally, *Experiences of individual contra family sessions* captures participants’ reflections on the different functions and strengths of individual versus family therapy sessions.

### 3.1 Perception of gaming as a problem

Most participants did not perceive themselves as having gaming disorder or problematic gaming behavior. When asked about in what way their gaming had been a problem for them, several explicitly stated that they did not believe their gaming had caused any significant issues in their lives. One participant explained:

*“Well, no, since it was never really a problem, I think. Because I would have thought it was a problem if I, like, didn’t go to school and just sat around, but I was going to school and all that.”* (P1)

Two participants identified their gaming as a problem from the start of the interview. One participant explicitly described himself as “being addicted to gaming” and stated that he recognized this because he had prioritized gaming over everything else for several years. Another participant described his gaming as excessive and believed that it negatively impacted his functioning in important areas of life, especially school.

*“I didn’t think I was addicted until after last year, [but looking back, I realize] I went home and played [ … ] from the moment I came home until bedtime, each and every day for nine years.”* (P3)

However, even though participants did not always characterize their gaming as problematic, most of them described it as having negative consequences in their daily life. Several participants reported that gaming affected their health, particularly their sleep which led them to be tired during the days, making it difficult for them to function in everyday life. One participant recalled how sleep deprivation had affected him:

*“I was at home gaming and I hadn’t slept for almost 24 hours, and when you’re gaming [you are exposed to] so much bright light, so I suffered some kind of, you know, epileptic seizure or something. That’s when my mom said, ‘Enough is enough.’”* (P1)

Half of the participants reported that gaming negatively affected their school performance, attendance, and grades. One participant reflected:

*“I never stopped [gaming], and it mainly affected my schoolwork and stuff like that. I just couldn’t focus on anything else than gaming because I was gaming all the time [….] I knew I had a problem because I could see my grades dropping every single day.”* (P7)

Many participants described conflicts with their parents in general and specifically about their gaming habits. They also mentioned social withdrawal as they spent more and more time gaming instead of engaging in other activities. They particularly mentioned that they choose to play instead of spending time with their family members.

Some participants proposed specific factors that they believed had contributed to their gaming habits. One participant explained that a traumatic event led to increased gaming. Having nothing else to do during recovery after an accident, it became a way to escape reality:

*“Well, I was in a traffic accident [a few years back], and I was in a wheelchair for almost the entire period from April to June or July. During that time, I spent about 90 percent of my time indoors, just playing games. And then it just continued. Even when I got better, I still had the urge to play.”* (P1)

Another participant suggested that neurodevelopmental conditions, such as autism and ADD, might increase vulnerability to excessive gaming. He linked his diagnosis to having greater difficulty in meeting daily responsibilities, which in turn influenced his gaming behavior:

*“Yes, I think that … well, many of these problems with gaming addiction can be strongly connected to diagnoses. It can be things like autism and such that contribute to becoming more addicted to gaming and so on … because I think that if I hadn’t had autism or ADD, I might not have become addicted to gaming, but I believe that’s the reason why I did.”* (P3)

### 3.2 Help-seeking and motivation for treatment

Most participants did not initiate treatment themselves and expressed low motivation or indifference at the start of treatment. One participant had experienced strong resistance to the treatment throughout the process. Others that had felt poorly motivated initially gradually developed motivation as they progressed in therapy. Only one participant reported being highly motivated from the start and maintaining that level of engagement throughout. The change in motivation was illustrated by one participant who at first was reluctant to participate in therapy because he doubted that he could be helped:

*“At first, I wasn’t really motivated. I think I even skipped [a few sessions] because, just because, you know [ … ]. I didn’t think that anything could help me. So I wasn’t very motivated to see, you know, like psychologists and stuff, because I didn’t believe that it would help. But after having went there a few times, I managed to feel more motivated and realized, ‘Okay, maybe this actually can help me.’ And in the end, it did.”* (P6)

Another participant, who acknowledged having a gaming-related problem, described how his motivation had grown with time from an initially low level when he felt that he was helped by the treatment:

*“I would say that at first, [my motivation] was very low. I went there just to see if it could help [ … ]. When I saw how I got closer to my parents and noticed the results, I would say that it grew, the motivation [to participate].”* (P7)

Many participants shared the experience of lacking a clear reason for seeking treatment and had a hard time articulating what exactly they needed help with. Even though all participants were directly asked in what ways their gaming had been a problem prior to treatment, and how their gaming had contributed to seeking professional help, several struggled to provide clear answers or reflect on these questions. It was not their own initiative to seek help, instead it was their parents or school that initiated the contact. For half of the participants, parents had initiated contact with the clinic due to concerns about health, sleep, or school attendance. In one case, a parent sought help after discovering that their child had lost money gambling which triggered a worry about gaming as well. One participant described that his mother sought help for his gaming, even though he didn’t feel he played much:

*“No, not that much [time spent gaming], it was my mom who sought [help]. [ … ] [she thought] that I played too much. But I wasn’t really allowed to play at all.”* (P5)

For two participants, school personnel introduced the idea of treatment, after concerns had been raised about poor attendance and academic performance.

*“It was actually both my mentors and my school nurse who introduced me to this … assessment. And it happened after my mentors raised concerns that I was never attending class. That was basically the reason I went there, to improve my grades and be more in class.”* (P7)

The fact that most participants did not initiate help-seeking themselves aligns with their low initial motivation and that they did not think that their gaming behavior was problematic.

### 3.3 Experiences and reflections of treatment

Participants’ experiences of the treatment include their overall satisfaction, what they thought was good, areas for improvement, and their perception of the treatment’s focus. While most participants described their experience as positive, they provided only limited reflections on what specifically worked well for them. Many found it difficult to elaborate on their thoughts about the treatment and often stated that they had nothing to add. It was common for participants to express general satisfaction, frequently mentioning that they liked the treatment, and saw no need for changes. This lack of clarity is reflected in one participant’s statement:

*“I think everything was good. It has helped me a lot”* (P5).

Several participants stated that the treatment addressed more than just gaming. One participant mentioned that gaming was discussed only briefly, while another stated that it was barely addressed at all. In contrast, some participants felt that a significant portion of their sessions focused on gaming-related issues, with some viewing it as a central focus while others experienced it as a secondary concern. But overall, participants described the treatment as being more focused on helping them manage other aspects of life, such as school, sleeping habits, relationships and increasing engagement in other activities. As one participant explained:

*“Yeah. No, not really. I guess it was more about going out and doing things and stuff like that, but not really [gaming]”* (P2)

Several participants expressed that they were satisfied with this approach, feeling that the balance between addressing gaming and other life factors was appropriate.

*“It [the treatment], doesn’t really focus on reducing your gaming, rather, it’s more about increasing other activities or things like that”* (P4)

Another described how the treatment initially focused on gaming but later shifted to his problems in school:

*“we started with the gaming, and it didn’t take long at all. It was about a week, and then we kind of … we came up with a plan, and we’ve just stuck to it. And now, we’ve almost only been talking about school”* (P5)

Feedback regarding specific aspects of the treatment that were perceived as less effective was limited as well, despite the interviewer explicitly asking about it. However, one participant emphasized the need to explore the underlying reasons behind excessive gaming and suggested that the therapist should take a more active role in identifying alternative activities to gaming. Another participant argued that the treatment had not focused on gaming until late in the treatment and would have wished it had done so more in the beginning instead. He also noted that long breaks between sessions led to a relapse into old habits, stating:

*“There was quite a long period when we didn’t meet, maybe a month or so, and … at the beginning of that period, it was fine, but I felt that the longer it went without a session, the more I returned to old habits, staying in my room all day and so on. And it felt like the longer we went without meeting, the worse it got”* (P7).

One participant was clearly negative about the treatment, stating that it had not been helpful at all. He felt it focused too much on reducing gaming and on tasks like cleaning his room and making his bed. He offered limited reflections beyond this, both in terms of what he disliked and what could have been improved.

*“Nothing helped. We agreed that I would go to bed at 9 p.m., and I do that.”* (P8)

Another participant noted that the treatment was not effective for him personally, and had a hard time suggesting how the treatment could be improved.

*“It’s not like there was anything wrong with the sessions themselves or anything like that. It was just mostly that I didn’t feel it worked for me, and in that case, it’s hard to say what could have been done differently”* (P4)

### 3.4 Changes following treatment

Reported changes from the treatment were primarily seen in three areas: gaming behavior, improvements in school and daily routines, and relationships with family members. Participants did vary in how their gaming behavior had changed after treatment. The changes in gaming behavior were often linked to changes in other important areas of life. Half of the participants reported that they played less than before, often describing their gaming as now being at a normal level, similar to their friends, which allowed them to engage in other activities.

*“Today, I still play, but not as much. Maybe around 20 hours a week, which I think is pretty normal.”* (P1)

For some, gaming activities became more structured, and they gained a better understanding of their gaming habits, which allowed them to manage their gaming more consciously:

*“I feel like I make an active choice to sit down at the computer. And I don’t really know what drives that decision, like what influences me in making it, but I do it … I think: “Now, now I’m sitting down at the computer because I want to play, not just because I’m at home, so I automatically sit down at the computer.”* (P7)

Many participants described improvements in other areas of life beyond gaming as a result of treatment. They mentioned better sleep, increased physical activity and more social interactions.

*“My sleep has improved a lot, and my gaming has improved in the sense that I play less. I have more time to do other things.”* (P3)

Better attendance and academic progress were reported by several participants. Two participants said that the treatment helped them return to school after being absent for several months.

*“Before, I didn’t go to school at all, but now I go almost every day. It has gotten better, and I’ll be starting high school in the fall.”* (P6)

Most participants reported improved relationships with family members and felt that their parents had developed a better understanding of their gaming habits. Some described that their parents had gained a more balanced view of gaming. Several participants described fewer conflicts at home related to gaming. One explained that while disagreements still occurred, they were less intense:

*“We used to have explosive fights at home with a lot of yelling and similar things, but it doesn’t feel like that happens much anymore, at least, it happens a lot less now”* (P7)

Even the participant who did think the treatment was not very helpful acknowledged that the conflicts in his family had decreased. While he remained critical, he recognized some improvement in his home environment:

*“Well, that’s the only thing, [the arguments] don’t happen as much anymore”* (P8)

It was common that the participants described how reducing their gaming and attending school more regularly improved the family climate, as it reduced parental stress and conflict at home. Another participant gained a deeper understanding of his parents’ concerns about gaming, which helped shift his perspective and resulted in a more cooperative approach.

*“Before, I saw my parents as the enemy, they were the ones trying to take gaming away from me. But through treatment, we’ve improved our relationship, and I understand why they were concerned. And they also understand why I played so much. So now, we understand each other better.”* (P7)

Not all participants experienced changes or lasting changes in their relationships. One participant described that he had always received strong support and understanding from his family, meaning there was no noticeable change in their relationships. This was the only participant who only had individual sessions and no family sessions. Another participant explained that his family dynamic improved during the treatment but later returned to the way it was before. He attributed this to the therapist’s guidance and suggestions on how to handle their relationship at home during the treatment. However, he noted that without the ongoing support of the therapist, his family relationships gradually returned to how they had been before treatment.

### 3.5 The therapeutic relationship and the therapist’s knowledge of gaming

The participants’ experiences of the therapeutic relationship centered around two key aspects: the quality of their relationship with the therapist and the therapist’s knowledge of gaming. Almost all participants described their relationship with the therapist as positive. They reported feeling comfortable, supported, and understood, and describing the therapist as easy to talk to, empathetic, and able to provide helpful insights. Some participants specifically mentioned that their therapist was a good match for them. One participant felt that the treatment and therapist were not necessarily bad but rather that it was not the right fit for him.

*“The therapist was easy to talk to and seemed to understand me in a different way than others do.”* (P2)

Almost all participants emphasized the importance of the therapist having knowledge about gaming and generally they perceived their therapists as being well-informed about gaming and its effects. Participants considered this to be beneficial for both the treatment process and the therapeutic relationship and emphasized that having a therapist with gaming-specific knowledge made it easier to feel comfortable and open in conversations. One participant expanded on what this would have meant for him in a previous psychological treatment:

*“If my therapist knew which game I played and asked me something like, ‘What rank are you?’ and things like that, it would have made it much easier to open up, because it feels good when they actually understand.* (P3)

Another participant similarly stressed how therapist knowledge positively impacted him treatment experience:

*“I think that if he hadn’t known so much, the treatment would have gone much worse, if he didn’t understand anything about what we were talking about. But he did, so I would say that was positive.”* (P4)

One participant downplayed the importance of therapist knowledge in their own case, since his treatment primarily focused on areas of life unrelated to gaming. Nonetheless, he still acknowledged that therapist expertise in gaming could be important for reaching other patients and making progress in treatment. Another participant emphasized the value of having a therapist who had personal experience with gaming-related difficulties, as this could improve understanding and connection. He argued that the therapist needs to have experienced this themselves to understand how it is and be able to help other people.

In contrast, one participant felt that their therapist lacked sufficient knowledge about gaming, which resulted in the participant having to explain and educate their therapist about video games. Even if he was critical of his therapist knowledge, he still, like the others said that it was important that the therapist knew about video-games and gaming.

*“He doesn’t even know. I have to explain to him what I play and how it works. And it feels like he doesn’t know much. He just tries to reduce [my gaming] without understanding how it works.”* (P8)

### 3.6 Individual contra family therapy sessions

Participants described individual and family sessions as serving different but complementary roles. In the individual sessions, participants had the opportunity to talk about their thoughts and feelings, while the family sessions were often described as emotionally challenging but important for increasing understanding and reducing conflict. As previously described, one participant had only individual sessions, while the rest had a combination of individual and family sessions. They expressed satisfaction both with the therapy format in general, and with the combination of individual and family sessions they received. Two participants explicitly wished for more individual sessions, while two others described the family sessions as the most beneficial part of their treatment.

The experience of individual therapy sessions was largely reported as positive, with participants describing them as an opportunity to focus on their personal experiences, emotions, and challenges. Some appreciated that these sessions allowed them to discuss issues they did not feel comfortable addressing with their parents. A few participants also found individual sessions useful for discussing practical matters. One participant noted that these sessions also served as a form of social training, helping him build confidence in speaking with a therapist and giving him the opportunity to practice expressing himself without his mother present. This points to the importance of being attentive to power dynamics in therapy, with adults unintentionally may overshadow the adolescent’s perspective.

*“when I sit alone with the psychologist, it becomes easier for me to speak my own mind. Because before, when I sat with my mom … my mom used to take over and answer for me.”* (P6)

The family sessions, on the other hand, were seen as important in improving family dynamics. Several participants described these sessions as challenging yet beneficial, with a particular focus on addressing conflicts and misunderstandings at home. Some reported that hearing their parents’ perspectives on their gaming behaviors helped them gain new insights into their family relationships.

*“The most helpful were probably the family sessions, where I got to take in what everyone else, or what they also felt about it. And to understand that I shouldn’t do it again…. but they were also the hardest.”* (P2)

Many participants described other differences between individual and family sessions as well, noting that family sessions tended to emphasize what was working, whereas individual sessions allowed for more open discussions about their problems.

## 4 Discussion

This qualitative study explored the experience of male adolescents undergoing a psychological treatment for gaming disorder. The participants generally had positive experiences, particularly appreciating the combination of family therapy and individual CBT sessions. They highlighted the value of a broad therapeutic focus that addressed not only gaming behaviors but also important areas of functioning such as their school situation and family relationships. Notably, most adolescents did not perceive their gaming behavior as their main problem; rather, they talked about their gaming in relationship to problems in other areas of functioning such as school, sleep and interpersonal relationships. The participants mostly did not view their gaming as their main problem. Instead, they tended to describe problems in other areas of life and the relationship between gaming and those problems. School problems emerged as particularly important reason for seeking treatment with several participants struggled with attendance. This aligns with previous research linking academic stress to problematic gaming (32). Notably, in individual cases, improvements in school appeared closely associated with reductions in problematic gaming, which further illustrates the interconnectedness of gaming behaviors with broader life challenges. Family conflicts were another frequently concern, and participants described family relationships as more distressing and central to their difficulties than the gaming itself. Again, this underscores how problematic gaming often is connected with broader life challenges.

These findings reflect the debate on whether gaming disorder should be considered a psychiatric diagnosis (46, 2). Kardefelt-Winther (46) argues that problematic gaming is better conceptualized as a coping mechanism or an external expression of underlying psychosocial distress rather than something which needs to be pathologized in itself. Similarly, Van Rooij et al. (2) question the usefulness of the diagnosis gaming disorder, arguing that negative consequences involving gaming can typically be explained in terms of broader psychosocial rather than gaming-specific factors. Our finding reflects this in some ways, as participants generally did not identify their gaming behavior as problematic in the clinical sense defined by ICD-11, but recognized that their gaming became problematic when it negatively impacted important areas of everyday functioning and interpersonal relationships. However, this does not mean that gaming is not a problem for these participants. Rather it shows the need for clinicians to be cautious about framing gaming as pathological, clinicians should explore, together with the patient, their perceived difficulties and how gaming relates to these challenges.

Overall, participants described their treatment experiences positively, especially with reference to the broad focus of the treatment and therapists’ knowledge about gaming. Many noted that the treatment not only targeted gaming behaviors but also addressed broader life areas, such as school, family relationships, and daily routines. While participants found it surprising that treatment for gaming disorder was not more focused on gaming itself, they considered this approach suitable for them personally, in that they recognized that their own gaming was connected to broader life difficulties.

This aligns with previous research showing that problematic gaming frequently co-occurs with psychiatric comorbidities, psychological distress, neurodevelopmental disorders (ADHD, ASD), suicidal ideation, impaired executive functioning, loneliness, social difficulties, sleep disturbances, and lower academic achievement (10, 11, 33–36). In line with Process-Based CBT principles proposed by Hayes and Hofmann (37), targeting the most accessible or influential symptoms within a network of interrelated symptoms can lead to improvements with minimal effort. Indeed, several participants noted significant improvements in school performance and family relationships, areas they valued highly, which suggests that positive change in one area of life often led to improvements in others.

The therapeutic relationship was mostly reported as positive, with most participants feeling understood and supported by their therapists. Therapists’ perceived knowledge of gaming and gaming culture appeared particularly important. Participants expressed that it was easier to engage with and open up to a therapists who appeared to understand the gaming world. Interestingly, therapists at the clinic often had only a basic understanding of gaming and no personal experience with problematic gaming, yet they were still perceived as knowledgeable and that this positively affected the treatment. This suggests that indicating that empathy, validation, and genuine interest might matter more than detailed expertise. This finding is consistent with research on the therapeutic alliance, which emphasizes that the effectiveness of psychological treatment is shaped not only by the therapist’s content expertise, but also by the patient’s experience of feeling accepted and supported, and by therapist qualities such as empathy, openness, and warmth (38, 39). These findings also align with research showing that cultural competence can increase the effectiveness of psychological treatments (40). Van Rooij, Schoenmakers (41) similarly argue that therapists who are familiar with digital culture are better positioned to build a strong therapeutic foundation with youth presenting with problematic internet use. On the other hand, clinicians’ own biases may create barriers: Ferguson (42) found that many clinicians hold negative beliefs about video games, especially when lacking direct experience of gaming, which can inadvertently affect how young clients feel perceived in therapy. This underlines the importance of therapists adopting a stance of curiosity rather than judgment. In line with this, even a basic understanding of gaming, when combined with empathy and non-stigmatizing attitudes, appears sufficient for building a strong therapeutic alliance with adolescents seeking help for gaming-related problems.

Participants described the combination of individual and family therapy sessions as beneficial. Family sessions were often experienced as more challenging. At the same time, participants reported that these sessions improved understanding and communication between them and their parents, reducing conflict and improving family relationships. Individual sessions provided an opportunity for the participants to speak more openly and address personal concerns, pointing to the importance of combining both therapeutic formats. Previous research on gaming disorder has primarily focused on CBT, but a recent meta-analysis suggested that, although more research is needed, incorporating family interventions into CBT may improve treatment effectiveness (18). The rationale for including family therapy is supported by evidence that gaming disorder develops within a familial context, where factors such as parent-child relationships play significant roles (43, 44). Problematic gamers tend to spend less time with parents, perceive parental relationships as more hostile, receive less affection, and describe their family climate as more problematic (15). Bonnaire, Liddle (14) argues for involving parents in treatment, highlighting that family relationships serve as protective factors and that parenting style influences adolescent development and emotional regulation. They particularly recommend interventions that help parents feel valued, encouraging sustained support and reducing critical communication. Our participants seem to agree with these ideas, repeatedly talking about the importance of improving communication with their parents and increasing mutual understanding.

### 4.1 Implications

The clinical implication that participants typically do not consider gaming their primary problem points to the need to broaden the therapeutic focus beyond gaming behavior to address underlying psychosocial issues. Although gaming may not represent adolescents’ main concern, it remains a central aspect of their daily lives and closely connected with their broader difficulties. Consequently, specialized clinics for gaming disorder are valuable, not because gaming itself should be the exclusive focus of intervention but because clinicians in specialized settings might be better equipped to recognize gaming as part of a broader network of interconnected symptoms, enabling interventions to prioritize areas where change will have the greatest impact. Specialized settings also offer adolescents a sense of their gaming being understood and that the gaming is not criticized, as they highlight how important it is that they feel that the clinicians have knowledge about gaming. Clinicians who possess knowledge about gaming can create greater patient engagement and strengthen the therapeutic alliance, even when treatment primarily targets areas other than gaming directly. Furthermore, integrating family therapy into treatment is important because it addresses the relational dynamics that contribute to gaming disorder. Strengthening family relationships not only reduces conflict but also enhances the adolescent’s sense of being supported and understood, which may be key to long-term improvements in both gaming behaviors and overall well-being.

### 4.2 Limitations

This study has several limitations that should be considered. On some topics, particularly when participants were asked to reflect on what was helpful in treatment or what could be improved, the data were limited. We believe this reflects the characteristics of the participant group, many of whom had low everyday functioning and struggled to reflect on these abstract topics. Therefore, we do not expect that a larger sample would have reduced this limitation. However, we have systematically missed the experiences of certain patients due to ethical considerations, as we excluded younger participants who were deemed too vulnerable to take part in the study.

Another limitation is that all participants were male. We made this choice deliberately, as only one female was available for interview at the time, and we wanted to reduce heterogeneity in the sample. The fact that the eligible sample pool only contained one female reflects the gender distribution typically seen in clinical settings, where gaming disorder predominantly affects males (9, 45). However, future research should explore the experiences of female patients and examine potential gender differences in experience of gaming disorder and the treatment of it.

Finally, as with all qualitative research, the aim was to generate a rich and detailed understanding rather than generalizable findings. The results should therefore be interpreted within the specific context of this study. Several steps were taken to enhance the validity of the study. Reflexivity was addressed by ensuring that the interviewer had no prior relationship with the participants, did not work at the clinic, and had limited prior knowledge of gaming disorder, which helped reduce potential bias during data collection. However, the first author, who participated in the analysis, was also a therapist at the clinic and had treated some of the participants. This insider perspective may have influenced the interpretation of the data. To strengthen the credibility of the findings, investigator triangulation was used: two researchers independently coded the data and discussed the analysis in regular meetings with the third author, a non-clinical mental health researcher, who contributed to reflexivity by challenging assumptions and offering an external perspective. This collaborative process helped ensure that the final categories were grounded in the material. A potential improvement would have been the inclusion of member checking, allowing participants to review and comment on the findings.

### 4.3 Conclusion

This study explored how adolescent patients perceive their gaming as a problem and their experiences of psychological treatment for gaming disorder. Most participants did not perceive their gaming behavior as their main problem. Instead, they had a tendency to describe their gaming in relation to other psychosocial difficulties, such as school, family relationships and routines. Rather than focusing exclusively on gaming behaviors, clinicians should explore how gaming relates to other areas of life. The participants were generally positive of this psychological treatment for gaming disorder, emphasizing both the broad therapeutic approach and the clinician’s understanding of gaming. Although therapists’ actual gaming expertise was limited, adolescents still felt understood and validated, stressing the importance of empathy and genuine interest in therapeutic practice over detailed expertise, as for most patients. Combining family therapy with individual CBT sessions was also reported as beneficial. Participants reported that family sessions improved mutual understanding, reduced conflict, and facilitated better family communication, which in turn improved their well-being. Thus, integrating family interventions into treatments for gaming disorder appears promising and clinically relevant achieve meaningful and sustained improvements.

## Data Availability

The raw data supporting the conclusions of this article will be made available by the authors, without undue reservation.

## References

[1] WHO. International statistical classification of diseases and related health problems (11th Revision), 11th ed. (2019), Geneva: World Health Organization.

[2] Van Rooij AJ Ferguson CJ Colder Carras M Kardefelt-Winther D Shi J Aarseth E. A weak scientific basis for gaming disorder: Let us err on the side of caution. J Behav addictions. (2018) 7:1–9. doi: 10.1556/2006.7.2018.19 PMC6035022 29529886

[3] Dullur P Starcevic V. Internet gaming disorder does not qualify as a mental disorder. Aust New Z J Psychiatry. (2018) 52:110–1. doi: 10.1177/0004867417741554 29119800

[4] Aarseth E Bean AM Boonen H Colder Carras M Coulson M Das D. Scholars’ open debate paper on the World Health Organization ICD-11 Gaming Disorder proposal. J Behav addictions. (2017) 6:267–70. doi: 10.1556/2006.5.2016.088 PMC5700734 28033714

[5] Billieux J King DL Higuchi S Achab S Bowden-Jones H Hao W. Functional impairment matters in the screening and diagnosis of gaming disorder: Commentary on: Scholars’ open debate paper on the World Health Organization ICD-11 Gaming Disorder proposal (Aarseth et al.). J Behav addictions. (2017) 6:285–9. doi: 10.1556/2006.6.2017.036 PMC5700712 28816514

[6] Müller KW Wölfling K. Both sides of the story: Addiction is not a pastime activity: Commentary on: Scholars’ open debate paper on the World Health Organization ICD-11 Gaming Disorder proposal (Aarseth et al. ). J Behav Addict. (2017) 6:118–20. doi: 10.1556/2006.6.2017.038 PMC5520131 28662617

[7] Mediemyndigheten. Gaming disorder – en kunskapsöversikt (Gaming disorder - a research overview). Stockholm: Mediemyndigheten. (2025). Available online at: https://www.mediemyndigheten.se/globalassets/rapporter-och-analyser/2024/gaming-disorder—en-kunskapsoversikt.pdf#page=10.72 (Accessed May 21, 2025).

[8] Rangmar J Thomée S. När datorspelandet blir problematiskt–en kunskapsöversikt om gaming disorder hos barn och unga. In: When gaming becomes problematic–a literature review about gaming disorder in children and youth Article in Swedish the County Administrative Board Sweden (2019) Stockholm.

[9] Stevens MW Dorstyn D Delfabbro PH King DL. Global prevalence of gaming disorder: A systematic review and meta-analysis. Aust New Z J Psychiatry. (2021) 55:553–68. doi: 10.1177/0004867420962851 33028074

[10] Richard J Temcheff CE Derevensky JL. Gaming disorder across the lifespan: A scoping review of longitudinal studies. Curr Addict Reports. (2020) 7:561–87. doi: 10.1007/s40429-020-00339-3

[11] Kristensen JH Pallesen S King DL Hysing M Erevik EK. Problematic gaming and sleep: a systematic review and meta-analysis. Front Psychiatry. (2021) 12:675237. doi: 10.3389/fpsyt.2021.675237 34163386 PMC8216490

[12] Gonzalez-Bueso V Santamaria JJ Fernandez D Merino L Montero E Ribas J. Association between internet gaming disorder or pathological video-game use and comorbid psychopathology: A comprehensive review. Int J Environ Res Public Health. (2018) 15. doi: 10.3390/ijerph15040668 PMC5923710 29614059

[13] Adams BLM Stavropoulos V Burleigh TL Liew LWL Beard CL Griffiths MD. Internet gaming disorder behaviors in emergent adulthood: a pilot study examining the interplay between anxiety and family cohesion. Int J Ment Health Addiction. (2018) 17:828–44. doi: 10.1007/s11469-018-9873-0

[14] Bonnaire C Liddle HA Har A Nielsen P Phan O. Why and how to include parents in the treatment of adolescents presenting Internet gaming disorder? J Behav Addict. (2019) 8:201–12. doi: 10.1556/2006.8.2019.27 PMC7044550 31146552

[15] Schneider LA King DL Delfabbro PH. Family factors in adolescent problematic Internet gaming: A systematic review. J Behav addictions. (2017) 6:321–33. doi: 10.1556/2006.6.2017.035 PMC5700711 28762279

[16] King DL Delfabbro PH Wu AMS Doh YY Kuss DJ Pallesen S. Treatment of Internet gaming disorder: An international systematic review and CONSORT evaluation. Clin Psychol Rev. (2017) 54:123–33. doi: 10.1016/j.cpr.2017.04.002 28458097

[17] Stevens MWR King DL Dorstyn D Delfabbro PH. Cognitive-behavioral therapy for Internet gaming disorder: A systematic review and meta-analysis. Clin Psychol Psychother. (2019) 26:191–203. doi: 10.1002/cpp.v26.2 30341981

[18] Kim J Lee S Lee D Shim S Balva D Choi KH. Psychological treatments for excessive gaming: a systematic review and meta-analysis. Sci Rep. (2022) 12:20485. doi: 10.1038/s41598-022-24523-9 36443408 PMC9705304

[19] King DL Wolfling K Potenza MN. Taking gaming disorder treatment to the next level. JAMA Psychiatry. (2020) 77:869–70. doi: 10.1001/jamapsychiatry.2020.1270 32492102

[20] Dong G Potenza MN. A cognitive-behavioral model of Internet gaming disorder: theoretical underpinnings and clinical implications. J Psychiatr Res. (2014) 58:7–11. doi: 10.1016/j.jpsychires.2014.07.005 25062755 PMC4448942

[21] André F Kapetanovic S Einarsson I Harvard ST Franzén L Möttus A. Relapse prevention therapy for internet gaming disorder in Swedish child and adolescent psychiatric clinics: a randomized controlled trial. Front Psychiatry. (2023) 14. doi: 10.3389/fpsyt.2023.1256413 PMC10623056 37928925

[22] Gurdal S Kapetanovic S Einarsson I Boson K Claesdotter-Knutsson E. Adolescents’ perceptions of a relapse prevention treatment for problematic gaming—a qualitative study. Healthcare (Basel). (2023) 11(17):2366. 37685400 10.3390/healthcare11172366 PMC10486974

[23] Werner M Kapetanovic S Nielsen M Gurdal S Andersson MJ Panican A. When the relationship is at stake: Parents’ perception of the relationship with a child with problematic gaming and their perceived need for support. Healthcare (Basel). (2024) 12(8):851. 38667613 10.3390/healthcare12080851 PMC11049846

[24] Torres-Rodríguez A Griffiths MD Carbonell X Oberst U. Treatment efficacy of a specialized psychotherapy program for Internet Gaming Disorder. J Behav Addictions. (2018) 7:939–52. doi: 10.1556/2006.7.2018.111 PMC6376389 30427213

[25] Nielsen P Christensen M Henderson C Liddle HA Croquette-Krokar M Favez N. Multidimensional family therapy reduces problematic gaming in adolescents: A randomised controlled trial. J Behav addictions. (2021) 10:234–43. doi: 10.1556/2006.2021.00022 PMC8996793 33905350

[26] Brandhorst I Lahres P Hanke S Batra A Renner T Barth G. Randomized controlled evaluation of a group-based training for parents of adolescents with gaming disorder or social network use disorder. Int J Environ Res Public Health. (2022) 20:272. doi: 10.3390/ijerph20010272 36612593 PMC9819552

[27] Marshall B Warburton WA Kangas M Sweller N. Internet gaming disorder (IGD) and smartphone addiction: parent intervention trial. Aust J Psychol. (2024) 76:2396961. doi: 10.1080/00049530.2024.2396961 PMC12218437 40666642

[28] Sandelowski M. Whatever happened to qualitative description? Res Nurs Health. (2000) 23:334–40. doi: 10.1002/1098-240X(200008)23:4<334::AID-NUR9>3.0.CO;2-G 10940958

[29] Sandelowski M. What’s in a name? Qualitative description revisited. Res Nurs Health. (2010) 33:77–84. doi: 10.1002/nur.20362 20014004

[30] Villamin P Lopez V Thapa DK Cleary M. A worked example of qualitative descriptive design: A step-by-step guide for novice and early career researchers. J Advanced Nurs. (2024). doi: 10.1111/jan.16481 PMC12271654 39382252

[31] Braun V Clarke V. Using thematic analysis in psychology. Qual Res Psychol. (2006) 3:77–101. doi: 10.1191/1478088706qp063oa

[32] Mihara S Higuchi S. Cross-sectional and longitudinal epidemiological studies of I nternet gaming disorder: A systematic review of the literature. Psychiatry Clin neurosciences. (2017) 71:425–44. doi: 10.1111/pcn.2017.71.issue-7 28436212

[33] Kim DJ Kim K Lee H-W Hong J-P Cho MJ Fava M. Internet game addiction, depression, and escape from negative emotions in adulthood: a nationwide community sample of Korea. J nervous Ment disease. (2017) 205:568–73. doi: 10.1097/NMD.0000000000000698 28598958

[34] Billieux J Potenza MN Maurage P Brevers D Brand M King DL. Cognitive factors associated with gaming disorder. Cogn Addict. (2020), 221–30. doi: 10.1016/B978-0-12-815298-0.00016-2

[35] Dullur P Krishnan V Diaz AM. A systematic review on the intersection of attention-deficit hyperactivity disorder and gaming disorder. J Psychiatr Res. (2021) 133:212–22. doi: 10.1016/j.jpsychires.2020.12.026 33360866

[36] Eltahir E Delfabbro PH King DL. Autism in relation to gaming disorder and Internet addiction: A systematic review. Comput Hum Behav. (2024) 162:108443. doi: 10.1016/j.chb.2024.108443

[37] Hayes SC Hofmann SG. Process-based CBT: The science and core clinical competencies of cognitive behavioral therapy. Oakland (CA): New Harbinger Publications (2018).

[38] Horvath AO Luborsky L. The role of the therapeutic alliance in psychotherapy. J consulting Clin Psychol. (1993) 61:561. doi: 10.1037/0022-006X.61.4.561 8370852

[39] Nienhuis JB Owen J Valentine JC Winkeljohn Black S Halford TC Parazak SE. Therapeutic alliance, empathy, and genuineness in individual adult psychotherapy: A meta-analytic review. Psychother Res. (2018) 28:593–605. doi: 10.1080/10503307.2016.1204023 27389666

[40] Soto A Smith TB Griner D Domenech Rodríguez M Bernal G. Cultural adaptations and therapist multicultural competence: Two meta-analytic reviews. J Clin Psychol. (2018) 74:1907–23. doi: 10.1002/jclp.2018.74.issue-11 30091201

[41] Van Rooij AJ Schoenmakers TM Vermulst AA Van Den Eijnden RJ Van De Mheen D. Online video game addiction: identification of addicted adolescent gamers. Addiction. (2011) 106:205–12. doi: 10.1111/j.1360-0443.2010.03104.x 20840209

[42] Ferguson CJ. Clinicians’ attitudes toward video games vary as a function of age, gender and negative beliefs about youth: A sociology of media research approach. Comput Hum Behavior. (2015) 52:379–86. doi: 10.1016/j.chb.2015.06.016

[43] Boson K Gurdal S Claesdotter-Knutsson E Kapetanovic S. Adolescent gaming and parent–child emotional closeness: Bivariate relationships in a longitudinal perspective. Curr Psychol. (2024) 43:19655–65. doi: 10.1007/s12144-024-05714-1

[44] Kapetanovic S Gurdal S Einarsson I Werner M André F Håkansson A. Relapse prevention therapy for problem gaming or internet gaming disorder in Swedish child and youth psychiatric clinics: protocol for a randomized controlled trial. JMIR Res Protoc. (2023) 12:e44318. doi: 10.2196/44318 36602846 PMC9853338

[45] André F Broman N Hakansson A Claesdotter-Knutsson E. Gaming addiction, problematic gaming and engaged gaming - Prevalence and associated characteristics. Addict Behav Rep. (2020) 12:100324. doi: 10.1016/j.abrep.2020.100324 33354616 PMC7744933

[46] Kardefelt-Winther D. A conceptual and methodological critique of internet addiction research: Towards a model of compensatory internet use. Computers in human behavior. (2014) 31:351–354.

[47] Bore P Nilsson S Andersson M Oehm K Attvall J Håkansson A. Effectiveness and acceptability of cognitive behavioral therapy and family therapy for gaming disorder: protocol for a nonrandomized intervention study of a novel psychological treatment. JMIR Res Protoc. (2024) 13(1):e56315. doi: 10.2196/56315 39151165 PMC11364953

