# Supplementary material for: Adolescents’ experiences of psychological treatment for gaming disorder: a qualitative study
Source: Front Psychiatry. 2025 Jun 3;16:1601851. doi: 10.3389/fpsyt.2025.1601851 (PMC12170625; doi:10.3389/fpsyt.2025.1601851)
Supplement: Supplementary file 1 [file DataSheet1.docx]

Supplementary Material

# Supplementary Data

**Interview guide**

***Introduction***

Thank you for participating! (Start with some small talk)

Brief presentation of the interviewer, the purpose of the interview, and how the data will be used. Estimated duration of the interview.

Explanation the ethical parts according to the information letter, including the right to withdraw and the option to decline any question.

Inform the participant that the interview will be recorded.

Any questions or concerns before we begin?

***Theme: How was gaming a problem for you***

Can you describe in what way gaming was a problem for you before the treatment?

What led you to seek treatment?

***Theme: Experience of the Treatment***

What did you think of the treatment for problematic gaming?

What part of the treatment was most helpful for you? – In what way?

Was there any particular theme/module that was helpful? – In what way?

What part of the treatment was least helpful for you? – In what way?

Was there any particular theme/module that was less helpful? – In what way?

***Theme: Focus of the Treatment***

What goals did you and your therapist set for the treatment?

In what way did you achieve the goals you set for the treatment?

To what extent do you feel the treatment focused on gaming itself?

Do you think it should have been different?

How did your motivation change before, during, and after the treatment?

How has the treatment changed the way you think about and understand your gaming behavior?

Were there any other problems, in yourself or your family, that were addressed during the treatment? Which ones?

And which do you think were most important to work on?

Did you receive any support related to school or work (e.g., from the municipality, employment services, or accommodations at school), and how did that help change your situation?

***Theme: Relationship with the Therapist***

How did you experience your relationship with your therapist?

How did you perceive your therapist’s knowledge about gaming?

How did your therapist’s knowledge (or lack thereof) about gaming affect your experience of the treatment?

Imagine you were a therapist conducting treatment for problematic gaming: what would you do differently?

***Theme: Has the Treatment Affected Relationships with Family Members?***

Has the treatment changed your relationship with your family members?

Do you think your family members’ view of your gaming has changed?

Have the conflicts at home around your gaming changed in any way?

***Theme: Individual vs. Family-Oriented Treatment***

What did your treatment look like in terms of who participated in the sessions?

Would you have wanted more or fewer individual sessions or family sessions?

If you had a combination of both, how did you experience the difference between the individual and family sessions?

What did you find good or less helpful about the different types of sessions?

***Concluding***

Is there anything else you’d like to add about the treatment?

Do you know where to turn if you were to feel in need of support from society?

Any final questions or thoughts before we end?

Information about where to turn for questions or additional support.

Thank you for your participation!
